# Association of polygenic score and the involvement of cholinergic and glutamatergic pathways with lithium treatment response in patients with bipolar disorder

**DOI:** 10.1038/s41380-023-02149-1

**Published:** 2023-07-11

**Authors:** Azmeraw T. Amare, Anbupalam Thalamuthu, Klaus Oliver Schubert, Janice M. Fullerton, Muktar Ahmed, Simon Hartmann, Sergi Papiol, Urs Heilbronner, Franziska Degenhardt, Fasil Tekola-Ayele, Liping Hou, Yi-Hsiang Hsu, Tatyana Shekhtman, Mazda Adli, Nirmala Akula, Kazufumi Akiyama, Raffaella Ardau, Bárbara Arias, Jean-Michel Aubry, Roland Hasler, Hélène Richard-Lepouriel, Nader Perroud, Lena Backlund, Abesh Kumar Bhattacharjee, Frank Bellivier, Antonio Benabarre, Susanne Bengesser, Joanna M. Biernacka, Armin Birner, Cynthia Marie-Claire, Pablo Cervantes, Hsi-Chung Chen, Caterina Chillotti, Sven Cichon, Cristiana Cruceanu, Piotr M. Czerski, Nina Dalkner, Maria Del Zompo, J. Raymond DePaulo, Bruno Étain, Stephane Jamain, Peter Falkai, Andreas J. Forstner, Louise Frisen, Mark A. Frye, Sébastien Gard, Julie S. Garnham, Fernando S. Goes, Maria Grigoroiu-Serbanescu, Andreas J. Fallgatter, Sophia Stegmaier, Thomas Ethofer, Silvia Biere, Kristiyana Petrova, Ceylan Schuster, Kristina Adorjan, Monika Budde, Maria Heilbronner, Janos L. Kalman, Mojtaba Oraki Kohshour, Daniela Reich-Erkelenz, Sabrina K. Schaupp, Eva C. Schulte, Fanny Senner, Thomas Vogl, Ion-George Anghelescu, Volker Arolt, Udo Dannlowski, Detlef Dietrich, Christian Figge, Markus Jäger, Fabian U. Lang, Georg Juckel, Carsten Konrad, Jens Reimer, Max Schmauß, Andrea Schmitt, Carsten Spitzer, Martin von Hagen, Jens Wiltfang, Jörg Zimmermann, Till F. M. Andlauer, Andre Fischer, Felix Bermpohl, Philipp Ritter, Silke Matura, Anna Gryaznova, Irina Falkenberg, Cüneyt Yildiz, Tilo Kircher, Julia Schmidt, Marius Koch, Kathrin Gade, Sarah Trost, Ida S. Haussleiter, Martin Lambert, Anja C. Rohenkohl, Vivien Kraft, Paul Grof, Ryota Hashimoto, Joanna Hauser, Stefan Herms, Per Hoffmann, Esther Jiménez, Jean-Pierre Kahn, Layla Kassem, Po-Hsiu Kuo, Tadafumi Kato, John Kelsoe, Sarah Kittel-Schneider, Ewa Ferensztajn-Rochowiak, Barbara König, Ichiro Kusumi, Gonzalo Laje, Mikael Landén, Catharina Lavebratt, Marion Leboyer, Susan G. Leckband, Alfonso Tortorella, Mirko Manchia, Lina Martinsson, Michael J. McCarthy, Susan McElroy, Francesc Colom, Vincent Millischer, Marina Mitjans, Francis M. Mondimore, Palmiero Monteleone, Caroline M. Nievergelt, Markus M. Nöthen, Tomas Novák, Claire O’Donovan, Norio Ozaki, Andrea Pfennig, Claudia Pisanu, James B. Potash, Andreas Reif, Eva Reininghaus, Guy A. Rouleau, Janusz K. Rybakowski, Martin Schalling, Peter R. Schofield, Barbara W. Schweizer, Giovanni Severino, Paul D. Shilling, Katzutaka Shimoda, Christian Simhandl, Claire M. Slaney, Alessio Squassina, Thomas Stamm, Pavla Stopkova, Mario Maj, Gustavo Turecki, Eduard Vieta, Julia Veeh, Stephanie H. Witt, Adam Wright, Peter P. Zandi, Philip B. Mitchell, Michael Bauer, Martin Alda, Marcella Rietschel, Francis J. McMahon, Thomas G. Schulze, Scott R. Clark, Bernhard T. Baune

**Affiliations:** 1 Discipline of Psychiatry, School of Medicine, University of Adelaide, Adelaide, SA Australia; 2 Centre for Healthy Brain Ageing (CHeBA), Discipline of Psychiatry and Mental Health, UNSW Medicine & Health, University of New South Wales, Sydney, Australia; 3 Northern Adelaide Local Health Network, Mental Health Services, Adelaide, SA Australia; 4 Neuroscience Research Australia, Sydney, NSW Australia; 5 School of Medical Sciences, University of New South Wales, Sydney, NSW Australia; 6 Institute of Psychiatric Phenomics and Genomics (IPPG), University Hospital, LMU Munich, Munich, Germany; 7 Department of Psychiatry and Psychotherapy, University Hospital, Ludwig-Maximilian-University Munich, Munich, Germany; 8 Institute of Human Genetics, University of Bonn, School of Medicine & University Hospital Bonn, Bonn, Germany; 9 Department of Child and Adolescent Psychiatry, Psychosomatics and Psychotherapy, LVR Klinikum Essen, University of Duisburg-Essen, Rheinische Kliniken, Essen, Germany; 10 Epidemiology Branch, Division of Population Health Research, Division of Intramural Research, Eunice Kennedy Shriver National Institute of Child Health and Human Development, National Institutes of Health, Bethesda, MD USA; 11 Intramural Research Program, National Institute of Mental Health, National Institutes of Health, US Department of Health & Human Services, Bethesda, MD USA; 12 HSL Institute for Aging Research, Harvard Medical School, Boston, MA USA; 13 Program for Quantitative Genomics, Harvard School of Public Health, Boston, MA USA; 14 Department of Psychiatry, University of California San Diego, San Diego, CA USA; 15 Department of Psychiatry and Psychotherapy, Charité - Universitätsmedizin Berlin, Campus Charité Mitte, Berlin, Germany; 16 Department of Biological Psychiatry and Neuroscience, Dokkyo Medical University School of Medicine, Mibu, Tochigi, Japan; 17 Unit of Clinical Pharmacology, Hospital University Agency of Cagliari, Cagliari, Italy; 18 Unitat de Zoologia i Antropologia Biològica (Dpt. Biologia Evolutiva, Ecologia i Ciències Ambientals), Facultat de Biologia and Institut de Biomedicina (IBUB), University of Barcelona, CIBERSAM, Barcelona, Spain; 19 Department of Psychiatry, Mood Disorders Unit, HUG - Geneva University Hospitals, Geneva, Switzerland; 20 Department of Molecular Medicine and Surgery, Karolinska Institute, Stockholm, Sweden; 21 Center for Molecular Medicine, Karolinska University Hospital, Stockholm, Sweden; 22 INSERM UMR-S 1144, Université Paris Cité, Département de Psychiatrie et de Médecine Addictologique, AP-HP, Groupe Hospitalier Saint-Louis-Lariboisière-F.Widal, Paris, France; 23 Bipolar and Depressive Disorders Program,, Institute of Neuroscience, Hospital Clinic, University of Barcelona, IDIBAPS, CIBERSAM, Barcelona, Catalonia Spain; 24 Department of Psychiatry and Psychotherapeutic Medicine, Research Unit for bipolar affective disorder, Medical University of Graz, Graz, Austria; 25 Department of Quantitative Health Sciences, Mayo Clinic, Rochester, MN USA; 26 Department of Psychiatry and Psychology, Mayo Clinic, Rochester, MN USA; 27 Université Paris Cité, Inserm, Optimisation Thérapeutique en Neuropsychopharmacologie, F-75006 Paris, France; 28 The Neuromodulation Unit, McGill University Health Centre, Montreal, Canada; 29 Department of Psychiatry & Center of Sleep Disorders, National Taiwan University Hospital, Taipei, Taiwan; 30 Department of Biomedicine, University Hospital Basel, Basel, Switzerland; 31 Institute of Neuroscience and Medicine (INM-1), Research Center Jülich, Jülich, Germany; 32 Douglas Mental Health University Institute, McGill University, Montreal, Canada; 33 Psychiatric Genetic Unit, Poznan University of Medical Sciences, Poznan, Poland; 34 Department of Biomedical Sciences, University of Cagliari, Cagliari, Italy; 35 Department of Psychiatry and Behavioral Sciences, Johns Hopkins University, Baltimore, MD USA; 36 Inserm U955, Translational Psychiatry laboratory, Fondation FondaMental, Créteil, France; 37 Max Planck Institute of Psychiatry, Munich, Germany; 38 Pôle de Psychiatrie Générale Universitaire, Hôpital Charles Perrens, Bordeaux, France; 39 Department of Psychiatry, Dalhousie University, Halifax, Nova Scotia Canada; 40 Biometric Psychiatric Genetics Research Unit, Alexandru Obregia Clinical Psychiatric Hospital, Bucharest, Romania; 41 University Department of Psychiatry and Psychotherapy Tuebingen, University of Tübingen, Tuebingen, Germany; 42 Department of General Psychiatry, University of Tuebingen, Tuebingen, Germany; 43 Department of Biomedical Resonance, University of Tuebingen, Tuebingen, Germany; 44 Department of Psychiatry, Psychotherapy and Psychosomatics, University Hospital of Frankfurt, Goethe University, Frankfurt, Germany; 45 Department of Immunology, Faculty of Medicine, Ahvaz Jundishapur University of Medical Sciences, Ahvaz, Iran; 46 Department of Psychiatry and Psychotherapy, Mental Health Institute Berlin, Berlin, Germany; 47 Institute for Translational Psychiatry, University of Münster, Münster, Germany; 48 AMEOS Clinical Center Hildesheim, Hildesheim, Germany; 49 Center for Systems Neuroscience (ZSN), Hannover, Germany; 50 Karl-Jaspers Clinic, European Medical School Oldenburg-Groningen, Oldenburg, 26160 Germany; 51 Department of Psychiatry II, Ulm University, Bezirkskrankenhaus Günzburg, Günzburg, Germany; 52 Department of Psychiatry, Ruhr University Bochum, LWL University Hospital, Bochum, Germany; 53 Department of Psychiatry and Psychotherapy, Agaplesion Diakonieklinikum, Rotenburg, Germany; 54 Department of Psychiatry and Psychotherapy, University Medical Center Hamburg-Eppendorf, Hamburg, Germany; 55 Department of Psychiatry, Health North Hospital Group, Bremen, Germany; 56 Department of Psychiatry and Psychotherapy, Bezirkskrankenhaus Augsburg, Augsburg, Germany; 57 Laboratory of Neuroscience (LIM27), Institute of Psychiatry, University of Sao Paulo, São Paulo, Brazil; 58 Department of Psychosomatic Medicine and Psychotherapy, University Medical Center Rostock, Rostock, Germany; 59 Clinic for Psychiatry and Psychotherapy, Clinical Center Werra-Meißner, Eschwege, Germany; 60 Department of Psychiatry and Psychotherapy, University Medical Center Göttingen, Göttingen, Germany; 61 German Center for Neurodegenerative Diseases (DZNE), Göttingen, Germany; 62 Psychiatrieverbund Oldenburger Land gGmbH, Karl-Jaspers-Klinik, Bad Zwischenahn, Germany; 63 Department of Neurology, University Hospital rechts der Isar, School of Medicine, Technical University of Munich, Munich, Germany; 64 Department of Psychiatry and Psychotherapy, University Hospital Carl Gustav Carus, Medical Faculty, Technische Universität Dresden, Dresden, Germany; 65 Department of Psychiatry and Psychotherapy, Philipps-University Marburg, Marburg, Germany; 66 Institute for Medical Informatics, University Medical Center Göttingen, Göttingen, Germany; 67 Mood Disorders Center of Ottawa, Ontario, Canada; 68 Department of Pathology of Mental Diseases, National Institute of Mental Health, National Center of Neurology and Psychiatry, 4-1-1 Ogawahigashi, Kodaira, Tokyo, 187-8553 Japan; 69 Service de Psychiatrie et Psychologie Clinique, Centre Psychothérapique de Nancy - Université de Lorraine, Nancy, France; 70 Department of Public Health & Institute of Epidemiology and Preventive Medicine, College of Public Health, National Taiwan University, Taipei, Taiwan; 71 Laboratory for Molecular Dynamics of Mental Disorders, RIKEN Brain Science Institute, Saitama, Japan; 72 Department of Psychiatry, Psychosomatic Medicine and Psychotherapy, University Hospital Frankfurt, Frankfurt, Germany; 73 Department of Psychiatry, Psychotherapy and Psychosomatic Medicine, University Hospital of Würzburg, Wurzburg, Germany; 74 Department of Adult Psychiatry, Poznan University of Medical Sciences, Poznan, Poland; 75 Department of Psychiatry and Psychotherapeutic Medicine, Landesklinikum Neunkirchen, Neunkirchen, Austria; 76 Department of Psychiatry, Hokkaido University Graduate School of Medicine, Sapporo, Japan; 77 Institute of Neuroscience and Physiology, the Sahlgrenska Academy at the Gothenburg University, Gothenburg, Sweden; 78 Department of Medical Epidemiology and Biostatistics, Karolinska Institutet, Stockholm, Sweden; 79 Inserm U955, Translational Psychiatry laboratory, Université Paris-Est-Créteil, Department of Psychiatry and Addictology of Mondor University Hospital, AP-HP, Fondation FondaMental, Créteil, France; 80 Office of Mental Health, VA San Diego Healthcare System, San Diego, CA USA; 81 Department of Psychiatry, University of Perugia, Perugia, Italy; 82 Section of Psychiatry, Department of Medical Sciences and Public Health, University of Cagliari, Cagliari, Italy; 83 Department of Pharmacology, Dalhousie University, Halifax, NS Canada; 84 Department of Clinical Neurosciences, Karolinska Institutet, Stockholm, Sweden; 85 Department of Psychiatry, VA San Diego Healthcare System, San Diego, CA USA; 86 Department of Psychiatry, Lindner Center of Hope / University of Cincinnati, Mason, OH USA; 87 Mental Health Research Group, IMIM-Hospital del Mar, Barcelona, Catalonia Spain; 88 Department of Genetics, Microbiology and Statistics, Faculty of Biology, University of Barcelona, Barcelona, Spain; 89 Department of Psychiatry and Psychotherapy, Medical University of Vienna, Vienna, Austria; 90 Institut de Biomedicina de la Universitat de Barcelona (IBUB), Barcelona, Spain; 91 Centro de Investigación Biomédica en Salud Mental (CIBERSAM), Instituto de Salud Carlos III, Madrid, Spain; 92 Neurosciences Section, Department of Medicine, Surgery and Dentistry “Scuola Medica Salernitana”, University of Salerno, Salerno, Italy; 93 Department of Psychiatry, University of Campania “Luigi Vanvitelli”, Naples, Italy; 94 National Institute of Mental Health, Klecany, Czech Republic; 95 Department of Psychiatry & Department of Child and Adolescent Psychiatry, Nagoya University Graduate School of Medicine, Nagoya, Japan; 96 Montreal Neurological Institute and Hospital, McGill University, Montreal, Canada; 97 Department of Psychiatry, Dokkyo Medical University School of Medicine, Mibu, Tochigi Japan; 98 Bipolar Center Wiener Neustadt, Sigmund Freud University, Medical Faculty, Vienna, Austria; 99 Department of Clinical Psychiatry and Psychotherapy, Brandenburg Medical School, Brandenburg, Germany; 100 Department of Genetic Epidemiology in Psychiatry, Central Institute of Mental Health, Medical Faculty Mannheim, University of Heidelberg, Mannheim, Germany; 101 School of Psychiatry, University of New South Wales, and Black Dog Institute, Sydney, Australia; 102 Department of Mental Health, Johns Hopkins Bloomberg School of Public Health, Baltimore, MD USA; 103 Department of Psychiatry and Behavioral Sciences, SUNY Upstate Medical University, Norton College of Medicine, Syracuse, NY USA; 104 Department of Psychiatry and Psychotherapy, University of Münster, Münster, Germany; 105 Department of Psychiatry, Melbourne Medical School, University of Melbourne, Parkville, VIC Australia; 106 The Florey Institute of Neuroscience and Mental Health, The University of Melbourne, Parkville, VIC Australia

**Keywords:** Genetics, Predictive markers, Bipolar disorder

## Abstract

Lithium is regarded as the first-line treatment for bipolar disorder (BD), a severe and disabling mental health disorder that affects about 1% of the population worldwide. Nevertheless, lithium is not consistently effective, with only 30% of patients showing a favorable response to treatment. To provide personalized treatment options for bipolar patients, it is essential to identify prediction biomarkers such as polygenic scores. In this study, we developed a polygenic score for lithium treatment response (Li^+^_PGS_) in patients with BD. To gain further insights into lithium’s possible molecular mechanism of action, we performed a genome-wide gene-based analysis. Using polygenic score modeling, via methods incorporating Bayesian regression and continuous shrinkage priors, Li^+^_PGS_ was developed in the International Consortium of Lithium Genetics cohort (ConLi^+^Gen: *N* = 2367) and replicated in the combined PsyCourse (*N* = 89) and BipoLife (*N* = 102) studies. The associations of Li^+^_PGS_ and lithium treatment response **—** defined in a continuous ALDA scale and a categorical outcome (good response vs. poor response) were tested using regression models, each adjusted for the covariates: age, sex, and the first four genetic principal components. Statistical significance was determined at *P* < 0.05. Li^+^_PGS_ was positively associated with lithium treatment response in the ConLi^+^Gen cohort, in both the categorical (*P* = 9.8 × 10^−^^12^, R^2^ = 1.9%) and continuous (*P* = 6.4 × 10^−^^9^, R^2^ = 2.6%) outcomes. Compared to bipolar patients in the 1^st^ decile of the risk distribution, individuals in the 10^th^ decile had 3.47-fold (95%CI: 2.22–5.47) higher odds of responding favorably to lithium. The results were replicated in the independent cohorts for the categorical treatment outcome (*P* = 3.9 × 10^−^^4^, R^2^ = 0.9%), but not for the continuous outcome (*P* = 0.13). Gene-based analyses revealed 36 candidate genes that are enriched in biological pathways controlled by glutamate and acetylcholine. Li^+^_PGS_ may be useful in the development of pharmacogenomic testing strategies by enabling a classification of bipolar patients according to their response to treatment.

## Introduction

Bipolar disorder (BD) is a severe and often disabling mental health disorder that affects more than 1% of the population worldwide and is characterized by recurrent episodes of depression and mania [1]. BD accounted for 9.3 million disability-adjusted life years (DALYs) in 2017, and imposes a significant social and economic burden on society and healthcare systems [2, 3]. BD is associated with a significant somatic and psychiatric comorbidity [1] and an increased risk of suicide [4].

Since the discovery of lithium’s mood-stabilizing property in 1949 [5], it has been widely used as a first-line therapy for patients with BD [6, 7]. Lithium is effective in treating acute episodes of illness and reduces the risk of future recurrences of mania and depression [8]. It has also been shown to reduce the risk of suicide [9]. Despite these merits, the efficacy of lithium is highly variable, with about 30% of treated patients showing a favorable response while more than 30% of them have no clinical response at all [8, 10]. Thus far, the causes and predictors of such heterogeneity in treatment response are insufficiently understood.

Genetic factors are thought to contribute, at least in part, to the large interindividual differences in response to lithium [10–15]. So far, only a few genetic studies have identified specific single nucleotide polymorphisms (SNPs) and candidate genes associated with patients’ response to lithium or treatment-related side effects [10, 11, 13–16]. Each employing a genome-wide association study (GWAS) approach, the Taiwan Bipolar Consortium found SNPs in the introns of *GADL1* associated with lithium treatment response [17], whereas the International Consortium on Lithium Genetics (ConLi^+^Gen) identified a locus on chromosome 21 [10], and a follow-up analysis uncovered additional variants within the human leukocyte antigen (HLA) region [14, 16]. Gene expression analysis of ConLi^+^Gen data also showed overexpression of genes involved in mitochondrial functioning in lithium responder patients, highlighting the electron transport chain as a potential target of lithium [18].

In our recent work, we applied a polygenic score (PGS) modeling approach and demonstrated associations between a poor response to lithium and a high genetic loading for schizophrenia (SCZ) [14], major depression (MD) [13], or a meta-PGS combining both SCZ and MD [15]. Machine-learning models that combined clinical variables with the PGS of SCZ and MD has further improved the prediction of lithium treatment response, explaining 13.7% of the variance [19].

Based on these previous results, translation of PGS testing into clinical practice requires the consideration of three important learnings. First, the PGS of a single phenotype (e.g., SCZ or MD) explains only a small proportion (<2%) of the variability to treatment response in patients with BD [13, 14], providing insufficient power for clinical use. Second, a meta-PGS from multiple related phenotypes has better predictive power than a PGS from a single phenotype [15], suggesting the need to explore additional biological markers, including additional PGSs, that can either independently or together with existing PGSs better predict lithium treatment response. Third, developing polygenic markers with *direct* pharmacogenomic implications is essential, for example, a PGS for lithium treatment response (Li^+^_PGS_), which is perhaps biologically more related to lithium’s pharmacological actions than PGSs built for other clinical phenotypes (i.e., SCZ or MD; that may indirectly influence treatment response or symptom severity, but do not index pharmacogenetic signatures per se).

Here, we developed a novel Li^+^_PGS_ for lithium treatment response and applied gene-based pathway analyses to identify molecular mechanisms impacted by genetic variation in response phenotypes. Findings may assist in optimizing and personalizing the selection of mood stabilizers in patients with BD, and may point to novel molecular targets for future drug development.

## Methods and materials

### Study samples

**Fig. 1:**
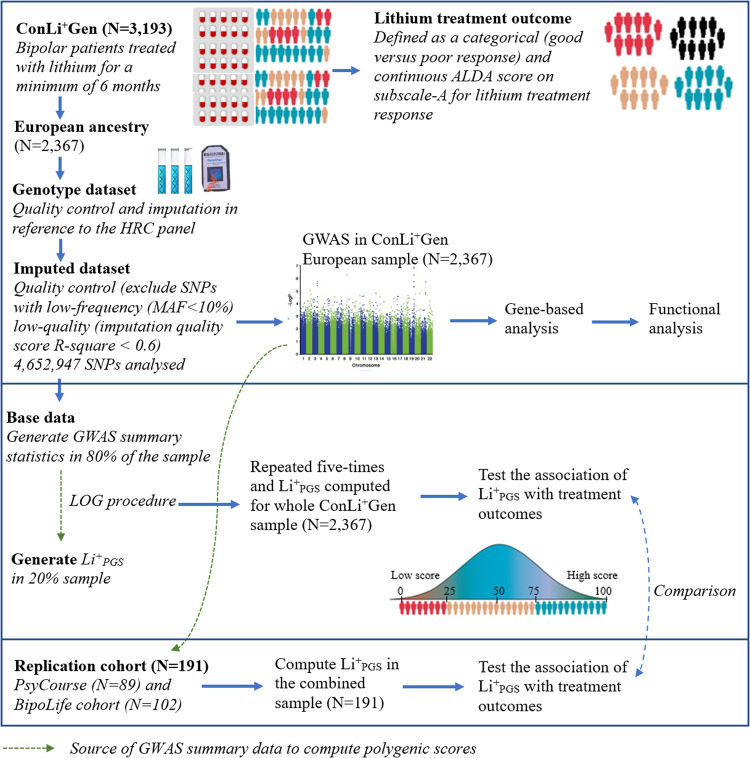
Overview of input datasets and steps of data analyses. ConLi^+^Gen = The International Consortium on Lithium Genetics, ALDA = Retrospective Criteria of Long-Term Treatment Response in Research Subjects with Bipolar Disorder scale, HRC = Haplotype Reference Consortium, SNPs = Single Nucleotide Polymorphisms, MAF=Minor Allele Frequency, GWAS = Genome Wide Association analysis, Li^+^_PGS_ = Polygenic score for lithium treatment response, LOG = Leave-one-group out procedure; PsyCourse = Pathomechanisms and Signature in the Longitudinal Course of Psychosis study and BipoLife = German research consortium for the study of bipolar disorder.

For this study, we obtained genetic and clinical data from the International Consortium on Lithium Genetics (ConLi^+^Gen: *N* = 2367), Pathomechanisms and Signature in the Longitudinal Course of Psychosis study (PsyCourse: *N* = 89), and BipoLife cohort (*N* = 102). Figure 1 shows the detailed steps of data analysis.

### Discovery cohort

*ConLi*^*+*^*Gen* is a global collaboration of scientists established to study the pharmacogenomics of lithium treatment in patients with BD [10]. In the current study, we analyzed the genome-wide genotype and clinical data of 2367 lithium-treated bipolar patients of European ancestry collected by 22 participating sites in 13 countries, including Australia (*n* = 122), Austria (*n* = 43), Czech Republic (*n* = 45), France (*n* = 210), Germany (*n* = 218), Italy (*n* = 255), Poland (*n* = 97), Romania (*n* = 152), Spain (*n* = 74), Sweden (*n* = 304), Switzerland (*n* = 57), Canada (*n* = 353) and the USA (*n* = 437) [10, 20].

### Replication cohort

To replicate Li^+^_PGS_ associations found in the discovery ConLi^+^Gen sample, we utilized datasets from PsyCourse and BipoLife where the study participants were of European ancestry. *PsyCourse* is a longitudinal multicenter study conducted from 2012 to 2019 in Germany and Austria, with up to four assessments at 6 monthly intervals. The study comprises 1320 patients from psychotic-to-affective spectrum, of which, datasets from 89 patients with BD who received lithium treatment were obtained for this study [21]. *BipoLife* is a multicenter cohort study, established to investigate the biological basis of BD and patients’ response to treatment and being conducted across ten university hospitals in Germany (Berlin, Bochum, Dresden, Frankfurt, Göttingen, Hamburg, Heidelberg, Marburg, Munich and Tübingen) and the medical informatics section of the University of Göttingen [22].

### Target outcome

In both discovery and replication cohorts, patient’s treatment response was assessed using the “Retrospective Criteria of Long-Term Treatment Response in Research Subjects with Bipolar Disorder” scale, also called the ALDA scale [10]. The target outcome “lithium treatment response” was defined in categorical and continuous scales among patients who had received lithium for a minimum of 6 months [10]. In both the discovery ConLi^+^Gen cohort and the replication cohorts (PsyCourse and BipoLife), a minimum of 6 months of lithium treatment follow-up was implemented as an inclusion criterion. This duration was chosen based on previous analyses of clinical trials, which established that a 6-month follow-up period is appropriate for assessing the minimum efficacy of lithium in patients with bipolar disorder [23]. Furthermore, clinical guidelines highly recommended to regularly monitor lithium levels during the initial six months of treatment, as this period is characterized by potential variability in lithium concentrations and an increased likelihood of side effects. After the six-month mark, stable lithium concentrations are typically achieved, allowing for an evaluation of the risk of toxicity and patients’ adherence to treatment. These factors ultimately influence the effectiveness of the treatment [24–26]. The detailed procedures of ALDA scale measurement and its validity are described elsewhere [13, 14, 20]. Briefly, the ALDA scale consists of two subscales: the A scale and the B scale. The A scale measures the response to lithium treatment on a continuum ranging from 0 to 10. Assessors evaluate the change in illness activity while the patient is receiving lithium, and the response is rated accordingly. The anchor points for the A scale range from no change or worsening (score = 0) to complete response, which includes no recurrences during adequate treatment, no residual symptoms, and full functional recovery (score = 10). On the other hand, the B scale describes five factors that could potentially confound the response to lithium treatment or the interpretation of its magnitude. These factors are the number and frequency of episodes before starting lithium (B1 and B2, respectively), the duration of lithium treatment (B3), adherence to the prescribed lithium regimen (B4), and the use of additional medications (B5). Each item on the B scale is rated on a scale of 0 to 2, with a higher B score indicating a lower level of confidence that any observed clinical improvement is solely due to lithium [27]. Once we calculated the total score as ‘A-score minus B-score and setting negative scores to zero’, the categorical (good versus poor) lithium treatment response was defined at a cut-off score of 7, where patients with a total score of 7 or higher were considered as “responders” [10]. The continuous outcome for lithium treatment response was defined on subscale-A, but patients with a total B score greater than 4 or who had missing data on the totals of ALDA subscale-A or B were excluded [10].

#### Genotyping, quality control and imputation

We obtained the genotype data assayed with different types of commercial SNP arrays across multiple cohorts [10, 21, 22] and applied a series of quality control (QC) procedures before and after imputation using PLINK [28]. First, SNPs that had a poor genotyping rate (<95%), strand ambiguity (A/T and C/G SNPs), a minor allele frequency (MAF) less than 1% or showed deviation from Hardy-Weinberg Equilibrium (*P* < 10^−^^6^) were removed. Then, individuals with low genotype rates (<95%), who had sex inconsistencies (between the documented and genotype-derived sex), and who were genetically related were excluded.

### Imputation

The genotype data passing QC were imputed on the Michigan server [24, 29] (https://imputationserver.sph.umich.edu) separately for each genotyping platform, using the Haplotype Reference Consortium (HRC) reference panel that consists of the largest available set (64,976 human haplotypes) of broadly European haplotypes at 39,235,157 SNPs [30]. For each cohort, imputation quality procedures were implemented to exclude SNPs of low-frequency (MAF < 10%) and low-quality (imputation quality score R-square < 0.6). From the imputed dosage score, genotype calls for the filtered SNPs were derived and common sets of 4,652,947 SNPs across the cohorts were merged using PLINK [28].

### Statistical analysis

We implemented polygenic score modeling, genome-wide SNP association, gene-based and functional analysis as described below.

#### Genome-wide SNP association analysis

Genome-wide SNP association analyses were performed on the binary lithium treatment response and continuous ALDA total score using logistic and linear regression models as implemented in PLINK software [28], respectively. Each analysis was adjusted for the covariates: age, sex, chip type and the first four genetic principal components (PCs). After careful examination of the Multidimensional (MD) plot, we observed that the first four PCs successfully captured and delineated any underlying population structure that could potentially influence the genetic association analyses. Consequently, these four PCs were incorporated as covariates in all association analyses. This approach aligns with the methodology employed by previous researchers who utilized the same dataset [10].

#### Polygenic score development

Using a polygenic score model constructed via Bayesian regression framework and continuous shrinkage (CS) prior on SNP effect sizes implemented in the PRS-CS software [31], we built Li^+^_PGS_ for individuals of European descent who participated in the ConLi^+^Gen study and replicated the findings in the combined PsyCourse and BipoLife datasets. Polygenic scores were computed using PRS-CS to infer posterior SNP effect sizes under continuous shrinkage (CS) using GWAS summary statistics and an external linkage disequilibrium (LD) reference panel. For the current analysis, the precomputed LD pattern of the 1000 Genomes European reference panel [32] and the discovery GWAS summary statistics were used to calculate PGS scores.

For the ConLi^+^Gen study, Li^+^_PGS_ was derived only for the European ancestry individuals (*n* = 2367) using a five-fold leave-one-group out (LOG) procedure [33] to remove discovery-target circularity. In each fold, 80% of the sample (*n* = 1893) was used to generate GWAS summary statistics that were used as discovery for PGS calculation in the 20% left-out target sample (*n* = 474). The procedure was repeated five times by selecting a non-overlapping set of 20% left-out samples to calculate PGS for the entire cohort. Finally, Li^+^_PGS_ was computed for the PsyCourse and BipoLife participants using ConLi^+^Gen’s GWAS summary statistics (discovery sample) generated from the full European cohort (*n* = 2367).

#### Polygenic score association analysis

To assess the association of Li^+^_PGS_ with lithium treatment response, a binary logistic regression model was applied for the binary outcome (good versus poor response to lithium treatment), and a Tobit analysis model (censored regression) was used for the continuous outcome (*ALDA total*) [34]. In addition, we divided the ConLi^+^Gen sample into deciles, ranging from the lowest polygenic load (1^st^ decile, reference group) to the highest polygenic load (10^th^ decile). Then, we compared BD patients in the higher polygenic load deciles (2^nd^–10^th^ deciles) with patients in the lowest polygenic load decile (1^st^ decile). In both the binary and continuous outcomes, the proportion of phenotypic variance explained by Li^+^_PGS_ was computed as the difference in R^2^ of the model fit with Li^+^_PGS_ plus covariates, compared to the model fit with only covariates. Each modeling analysis was adjusted for the covariates: age, sex, and the first four genetic PCs, and statistical significance was set at *p* < 0.05.

#### Gene-based and functional analysis

The gene-based analysis was based on summary statistics generated through genome-wide SNP association analysis of the full European ConLi^+^Gen sample (*n* = 2367) and employed MAGMA (Multi-marker Analysis of GenoMic Annotation) [35], a tool that uses a multiple regression approach to incorporate LD between markers and to detect multi-marker effects.

To explore the biological context of the genes discovered from the gene-based analysis, a pathway analysis was implemented using PANTHER (Protein ANalysis THrough Evolutionary Relationships; http://pantherdb.org/) classification system. PANTHER is designed to classify proteins (and their genes) into biological pathways [36]. To prepare the input genes for PANTHER, we selected genes that showed gene-level association with lithium treatment response (either with the categorical or continuous outcome) at MAGMA adjusted *p*-value < 0.001. This list of genes was entered into PANTHER version-17 which compares the proportion of input genes mapping to a biological pathway to the reference gene list from its databases. Molecular relationships previously experimentally observed in Homo sapiens (human) were included. The significance of the overrepresented PANTHER pathways was determined using Fisher’s exact test and later adjusted for multiple testing using the Bonferroni correction method. Significant associations were defined at *p*-value < 0.05.

## Results

### Sample characteristics

The discovery analysis consisted of ConLi^+^Gen data obtained from 2,367 bipolar patients of European ancestry who had undergone lithium treatment for at least six months. The mean (sd) age of the patients was 47.5(13.9) years and 1,369 (57.8%) were female. In all, 660 (27.9%) of patients had a good response to lithium treatment (ALDA score ≥7). The mean (sd) ALDA score for ConLi^+^Gen participants was 4.1 (3.1). Among 2362 patients who underwent assessment for the type of bipolar diagnosis, the majority (80.0%) were diagnosed with type 1 bipolar disorder. These patients also presented with comorbid conditions such as psychosis, alcohol dependence, panic disorder, and obsessive-compulsive disorder. Of the 438 patients assessed for possible side effects related to lithium treatment, 153(34.9%) of them reported experiencing at least one of the following: nausea, vertigo, polyuria, diarrhea, hypothyroidism, loss of libido, EEG abnormalities, increased thirst, dermal problems, weight gain, and strangury. The replication analysis was based on a combination of the PsyCourse and BipoLife datasets (*N* = 191), whose mean (sd) age was 49.1(13.0) years. Of the 191 patients with BD, 48(25.1%) had a good response to lithium. This replication cohort exhibits similar characteristics to the discovery sample in terms of the type of bipolar disorder, comorbidities, and patients’ reports of lithium treatment side effects (Table 1).

**Table 1:** The characteristics of patients with BD and lithium treatment outcomes.

Characteristics BD patients	ConLi^+^Gen	PsyCourse and BipoLife combined
*N* = 2558	*N* = 2,367	*N* = 191
Good responders to lithium defined as ALDA total score ≥ 7, *N* (%)	660 (27.9%)	48 (25.1%)
Mean (se) total ALDA score	4.12 (3.15)	4.3 (2.9)
Country of origin	***N*** **(%)**	***N*** **(%)**
Australia	122 (5.2)	
Austria	43 (1.8)	
Canada	353 (14.9)	
Czech Republic	45 (1.9)	
France	210 (8.9)	
Germany	218 (9.2)	191 (100%)
Italy	255 (10.8)	
Poland	97 (4.1)	
Romania	152 (6.4)	
Spain	74 (3.1)	
Sweden	304 (12.8)	
Switzerland	57 (2.4)	
USA	437 (18.5)	
Age at interview, mean (sd)	47.5 (13.9)	49.1 (13.0)
Sex, Female, *N* (%)	1369 (57.8)	84 (44.0%)
*Type of bipolar diagnosis, N (%)*	2362 (99.8)	89 (46.6)
Bipolar type I	1890(80.0)	75(84.3)
Bipolar type II	440(18.6)	14(15.7)
Bipolar type III	7(0.3)	
Bipolar not specified	7(0.3)	
Schizoaffective bipolar disorder	18(0.8)	
*Comorbidity*	***N***^***¥***^ **(% with)**	***N***^***¥***^ **(% with)**
Psychosis	2096 (53.2)	103 (3.9)
Alcohol dependence	933 (18.0)	102 (5.9)
Panic disorder	926 (13.6)	102 (8.8)
Obsessive-compulsive disorder	923 (5.2)	103 (2.9)
Suicidal ideation	-	98 (66.3)
Lithium side effects	438 (34.9)	102 (83.3)

*BD* Bipolar disorder, *N* Number of individuals in each group, *sd* Standard deviation, *se* Standard error.

N^¥^ refers to the number of individuals assessed for comorbidities, suicidal ideation or lithium side effects.

### Associations of Li^+^_PGS_ with lithium treatment response in bipolar patients

**Fig. 2:**
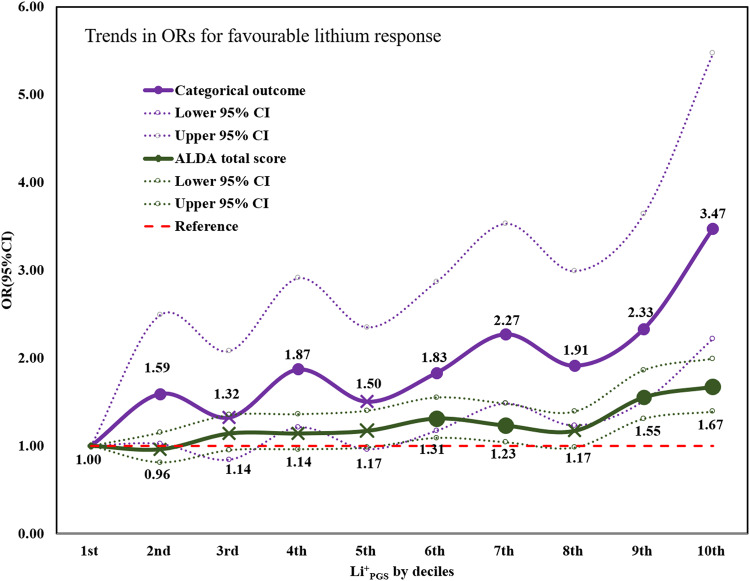
Trends in the odds ratios (ORs) for favourable treatment response to lithium for patients with bipolar disorder in the higher genetic loading for lithium responsive variants, (2^nd^ to 10^th^ deciles) compared with patients in the lowest (1^st^ decile) of genetic loading for lithium response (*n* = 2367). The X mark on the line plot indicates that the association is not statistically significant at that decile. OR Odds ratio, CI Confidence interval, Li^+^_PGS_ Polygenic score for lithium treatment response.

Using ConLi^+^Gen data, we found statistically significant associations between Li^+^_PGS_ and lithium treatment response — both in the categorical (*P* = 9.8 × 10^−^^12^, R^2^ = 1.9%) and continuous (*P* = 6.4 × 10^−^^9^, R^2^ = 2.6%) outcomes. Li^+^_PGS_ was positively associated with response to lithium treatment, with an adjusted odds ratio (OR) [95%CI]) of **1.39** [1.26, 1.54]. In other words, BD patients who carry a higher genetic loading for lithium responsive genetic variants, measured using the Li^+^_PGS_, have higher odds of favorable lithium treatment response, compared to patients carrying a low Li^+^_PGS_ load. Table 2 shows the association results of Li^+^_PGS_ and lithium treatment response in categorical and continuous outcomes. The odds of a favorable treatment response increased as the Li^+^_PGS_ increased, ranging from 1.59 fold [95%CI: 1.02–2.49] at the 2^nd^ decile to 3.47 fold [95%CI: 2.22–5.47] at 10^th^ decile, compared to the reference Li^+^_PGS_ at the 1^st^ decile (Table 2). While there was an increasing trend in the odds of lithium treatment response across the deciles, the most significant prediction contrast was found at the ‘extremes’ (1^st^ and 10^th^ decile) which comprised of ~20% of the total cohort (Fig. 2). A replication PGS analysis in the combined PsyCourse and BipoLife samples found a statistically significant association of Li^+^_PGS_ with the categorical lithium treatment response (*P* = 3.9 × 10^−^^4^, R^2^ = 0.9%), but not with the continuous outcome (*P* = 0.13).

**Table 2:** The association of PGS for lithium variants and treatment response to lithium in patients with BD at different sample splits.

Sample split	*N*	Categorical outcome, OR (95%CI)		Continuous outcome: ALDA total score, OR (95%CI)	
ConLi^+^Gen	2367	unadjusted	adjusted	unadjusted	adjusted^¥^
80%/20%	2083/284	1.31(1.19,1.43)	1.39(1.26, 1.54) ^¥^	1.15(1.11, 1.20)	1.17(1.13, 1.22)
Li^+^_PGS_ by decile	^§^R/N				
First (lowest score)	44/236	1[Reference]	1[Reference]^¥^	1[Reference]	1[Reference]
Second	60/237	1.48(0.96, 2.30)	1.59(1.02, 2.49)	0.94(0.79,1.12)	0.96(0.81,1.15)
Third	54/237	1.29(0.82, 2.02)	1.32(0.84, 2.08)	1.07(0.90,1.28)	1.14(0.95,1.35)
Fourth	70/237	1.83(1.19, 2.83)	1.87(1.21, 2.91)	1.09(0.92,1.31)	1.14(0.96,1.36)
Fifth	59/236	1.45(0.94, 2.27)	1.50(0.96, 2.35)	1.12(0.93,1.34)	1.17(0.98,1.40)
Sixth	62/237	1.55(1.00, 2.40)	1.83(1.17, 2.87)	1.22(1.02,1.46)	1.31(1.09,1.55)
Seventh	76/237	2.06(1.35, 3.17)	2.27(1.48, 3.53)	1.15(0.96,1.38)	1.23(1.04,1.48)
Eighth	68/237	1.76(1.14, 2.72)	1.91(1.23, 2.99)	1.12(0.93,1.34)	1.17(0.98,1.39)
Nineth	78/237	2.14(1.41, 3.29)	2.33(1.51, 3.64)	1.45(1.21,1.72)	1.55(1.31,1.86)
Tenth (highest score)	89/236	2.64(1.74, 4.05)	3.47(2.22, 5.47)	1.52(1.27,1.82)	1.67(1.39,1.99)

The reference decile (1^st^ decile) is the PGS category with the lowest polygenic load for lithium variants. OR (95%CI) for the continuous outcome: ALDA total score is calculated as the exponent of beta coefficient from the linear regression model.

^§^R/N: number of lithium responders versus total in that decile; ^¥^ adjusted for age, sex and 4-genetic principal components, OR: odds ratio.

### Genome-wide association, gene-based and functional analysis

**Fig. 3:**
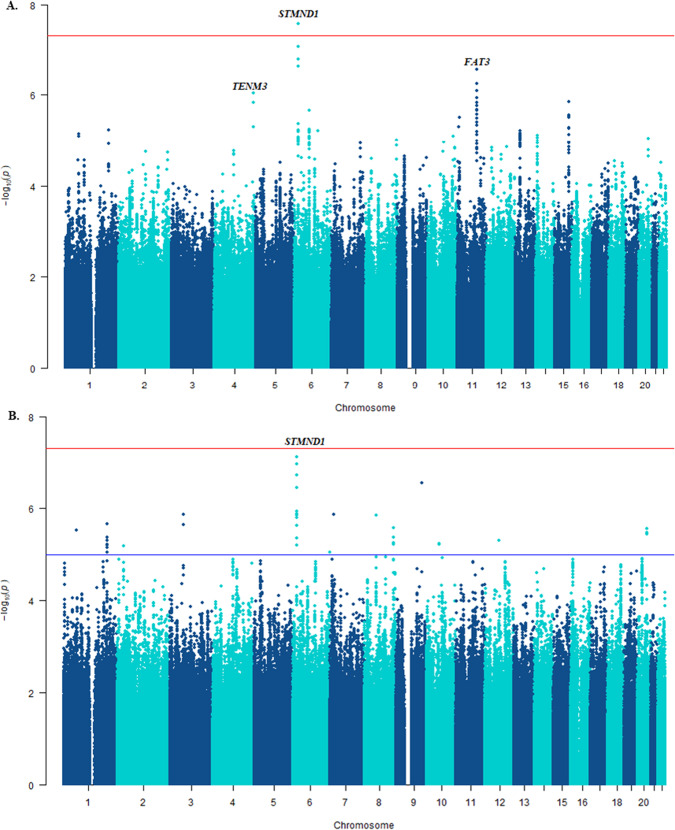
Manhattan plots showing the SNP-based GWAS results of lithium treatment response in patients with bipolar disorder. **A** In the categorical outcome and (**B**) continuous scale, highlighting the loci that showed genome-wide significance (orange).The −log10 (*p*-value) is plotted against the physical position of each SNP on each chromosome. The threshold for genome-wide significance (*p*-value < 5 × 10^–8^) is indicated by the red dotted horizontal line.

After re-imputing the ConLi^+^Gen data in reference to the latest HRC genomes, we conducted GWASs on lithium response, both in categorical and continuous outcomes. This GWAS analysis identified a single locus with lead SNP rs9396756 located near the stathmin domain containing 1 (*STMND1)* gene that reached genome-wide significance for association with the categorical outcome (*P* = 2.7 × 10^−^^8^) and showed a suggestive association with the continuous ALDA score (*P* = 7.6 × 10^−^^8^) (Fig. 3). A follow-up gene-based analysis of the newly derived ConLi^+^Gen GWAS summary statistics found 36 candidate genes likely associated with lithium treatment response — assessed in either continuous or categorical outcomes (*P* < 0.001). In silico functional analysis of the 36 genes revealed enriched biological pathways including the muscarinic acetylcholine receptors 1 and 3 (*P*-value corrected for multiple testing = 0.026) and metabotropic glutamate receptor group III pathway (*P* = 0.043). These genes and pathways may have an impact on clinical response to lithium treatment and be potential molecular targets for lithium (Supplementary Figure 1 and Table 1).

## Discussion

This study presents findings from a comprehensive analysis of genetic and clinical data on lithium treatment response that involved the development of a polygenic score for lithium treatment response (Li^+^_PGS_), genome-wide SNP association and gene-based and functional analyses.

Since the publication of the first GWAS report by the ConLi^+^Gen team [10], two landmark studies that independently showed the negative association of PGSs for SCZ and MD with lithium treatment response have been published [13–15]. The first study found that 10% of bipolar patients with the lowest polygenic load for SCZ were 3.46 times more responsive to lithium compared to 10% of patients with the highest genetic load for SCZ [14, 15]. Similarly, in the second study, 10% of patients who had the lowest genetic loading for MD were 1.54 times more responsive to lithium than 10% of patients with the highest genetic loading for MD [13, 15]. Nevertheless, each of these PGSs accounts for <2% of the total variance to lithium treatment response [13], suggesting the need to explore additional biological traits that can either independently, or in concert with existing PGSs better predict lithium response. Moreover, the previous PGSs for SCZ and MD are difficult to interpret in a pharmacogenomic context, making the development of a specific lithium response PGS necessary, which is assumed to be more likely to be associated with lithium treatment response and perhaps is biologically more related to lithium’s pharmacological actions.

In this novel study, we constructed a PGS for lithium response-Li^+^_PGS,_ a biological marker of direct pharmacogenomic relevance, and showed a positive relationship between a high genetic loading for lithium treatment response variants and long-term therapeutic response to lithium in patients with BD. We demonstrated that bipolar patients at the extreme tail end of the distribution have the strongest association, i.e. 10% of patients who carry high genetic loading for lithium responsive variants (10^th^ decile) were 3.47 times more likely to respond to lithium compared to 10% of those with the lowest Li^+^_PGS_ (1^st^ decile). These results indicated that Li^+^_PGS_ has the potential to help stratify bipolar patients according to predicted lithium response.

In a GWAS of lithium treatment response, we identified a locus near the *STMND1* gene, which encodes for proteins known to be involved in neuron projection development, and active in neuron junctions and cytoplasm. Previous analysis that employed the 1000 Genomes Project reference panel for imputation reported a suggestive association between genetic variants within the *STMND1* gene and lithium treatment response [10].

Using our newly derived ConLi^+^Gen GWASs summary statistics as an input, we then carried out a gene-based analysis where several genetic variations were examined together for their association with lithium treatment response [35]. This approach found 36 potential target genes for lithium treatment that are enriched in the muscarinic acetylcholine receptors (mAChRs) 1 and 3 and the metabotropic glutamate receptor group III signaling pathways — well characterized biological pathways modulated by the most abundant neurotransmitters in the brain (glutamate and acetylcholine).

Acetylcholine is the central regulator of the mAChRs signaling pathways, which are subfamily of G protein-coupled receptor complexes located in the cell membranes of neurons and other cells that regulate fundamental functions of the central and peripheral nervous system including acting as the main end-receptor stimulated by acetylcholine released from postganglionic fibers in the parasympathetic nervous system [37]. The muscarinic antagonist scopolamine has antidepressant activity, while physostigmine, a cholinesterase inhibitor induces depressive symptoms, suggesting muscarinic receptors may play a role, not only in the pathogenesis of mood disorders, but also as therapeutic targets [38]. M1 and 3 receptors are localized in the cortex, hippocampus and substantia nigra and are known to activate protein kinase C (PKC), causing post-synaptic excitation. PKC is thought to be central in the molecular pathogenesis of BD.

Glutamate, the primary excitatory neurotransmitter in the central nervous system (CNS), exerts neuromodulatory actions via the activation of metabotropic glutamate (mGlu), a type of glutamate receptor that modulates synaptic transmission and neuronal excitability throughout the central nervous system [39]. Group III metabotropic glutamate receptors are largely presynaptically localized and downregulate neurotransmitter release from presynaptic terminals directly or indirectly. These receptors have a prominent expression in the brain, especially in the region of the hippocampus, and can lead to the inhibition of the cAMP cascade which is critical for the maintenance of long-term synaptic plasticity [40]. Growing evidence indicates that abnormalities in the glutamatergic system are implicated in the pathogenesis and treatment of mental health disorders [41] including BD [42, 43], SCZ [44], neurodevelopmental disorders [45], Huntington’s disease [46] and Alzheimer’s disease [47]. Studies have reported SNPs of the mGluRs system associated with BD [48], and in animal studies, lithium was found to alter intracellular calcium by modulating the activity of the metabotropic glutamatergic receptor system [49]. To summarise, findings from the genome-wide SNP association, gene-based and functional analysis highlight the possibility that mechanisms involving glutamate and acetylcholine signaling pathways might influence the therapeutic effects of lithium in patients with BD. Modulation of these pathways through genetic variants may disrupt or enhance lithium’s clinical effectiveness.

Our study has some limitations. First, while our findings were replicated in an independent small size sample, the fact that it was replicated in the binary outcome, but not in the continuous outcome indicates the need for a larger replication cohort. Second, because Li^+^_PGS_ was developed and evaluated in European-ancestry populations, the findings should be replicated in a multi-ethnic population to gauge generalizability. Furthermore, the risks and benefits of predictive models consisting of Li^+^_PGS_ should be evaluated in prospective studies. Third, Li^+^_PGS_ only explains about 2% of response variance in our cohort, and as such is comparable to PGSs from other phenotypes (SCZ, MDD) that have shown an association with treatment outcomes. On their own, these PGSs are not suited to clinical pharmacogenomic testing as they would not predict treatment response prospectively in individual patients. Prediction models combining Li^+^_PGS_ with other PGSs [13, 14] and clinical characteristics [19] may improve the clinical utility of PGSs. Such models would then need to be tested in prospective studies and clinical trials. Forth, studies have shown that approaches to phenotyping of lithium treatment response can be improved using advanced methods such as machine learning [19]. Employing a more precise phenotype definition may result in the identification of novel candidate genes implicated in lithium treatment response and ultimately the development of more informative Li^+^_PGS_. Fifth, the current analysis did not include important covariates such as medication dose, information on lithium blood levels, side effects, and the use of concomitant medications (such as Angiotensin-converting enzyme (ACE) inhibitors, diuretics, Non-steroidal anti-inflammatory drugs (NSAIDs)), which can potentially influence lithium clearance and treatment response [50]. Moreover, maintaining therapeutic blood levels is crucial to achieving treatment response with limited side effects in lithium therapy [50]. Lithium possesses a narrow therapeutic index, meaning that there is a relatively small margin between an effective dose and a potentially toxic one. Typically, lithium levels are initially monitored more frequently (weekly or biweekly) during the initiation or adjustment phase of medication, and then less frequently (every 3 to 6 months) once stable therapeutic levels are achieved. While the duration of lithium treatment and the use of certain psychiatric medications (antidepressants, antipsychotics, mood stabilizers) were assessed as part of the B scale measure of ALDA score, information on the specific dosage, medication blood level and the use of concomitant medications were not available in the ConLi^+^Gen dataset, and thus, they were not considered in our analyses. The inclusion of these pharmacogenomic covariates could provide stronger evidence and should be considered in future research.

In conclusion, we developed a unique lithium treatment response polygenic score (Li^+^_PGS_) that showed a positive association with better lithium treatment response in patients with BD. Our gene-based and functional analyses build upon the findings from existing molecular studies by linking lithium treatment response with muscarinic acetylcholine receptor signaling and metabotropic glutamate receptor pathways. Further pharmacological evaluation of these pathways in the context of BD and mood stabilizing treatments may prove fruitful.

### Supplementary information

Supplementary Figure and Table
