# Supplementary material for: Association of polygenic score and the involvement of cholinergic and glutamatergic pathways with lithium treatment response in patients with bipolar disorder
Source: Mol Psychiatry. 2023 Jul 11;28(12):5251–61. doi: 10.1038/s41380-023-02149-1 (PMC11041653; doi:10.1038/s41380-023-02149-1)
Supplement: Supplementary file 1 — Supplementary Figure and Table [file 41380_2023_2149_MOESM1_ESM.docx]

**Supplementary Figure 1 and Table 1**


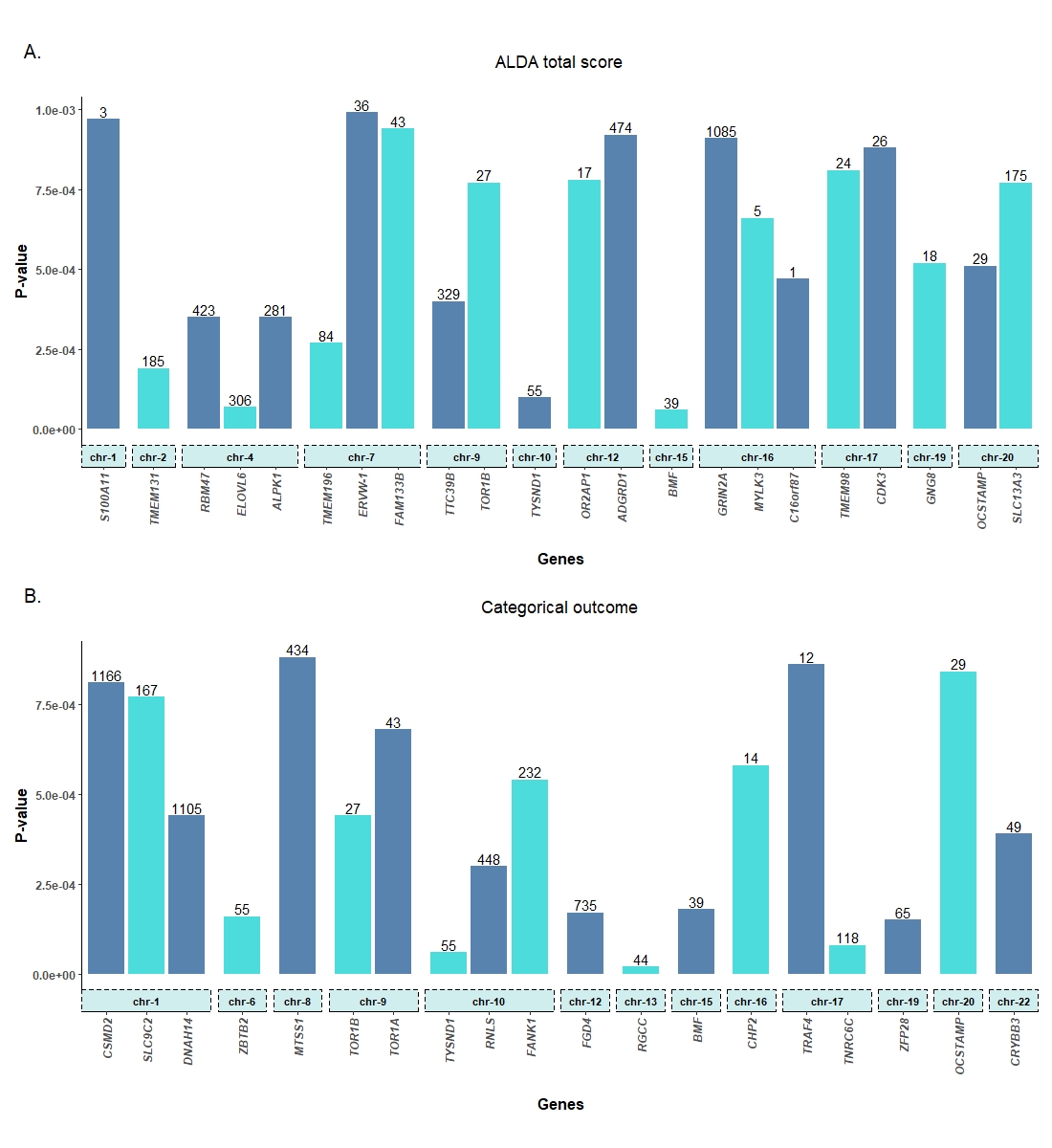


**Supplementary Figure 1.** Top candidate genes from MAGMA (Multi-marker Analysis of GenoMic Annotation) gene-based analysis[^1^](#_ENREF_1) (P<0.001). The plot shows on the Y-axis the gene-level association P-values for each gene positioned along the X-axis in genomic order by chromosomal position. The upper panel (A) shows the candidate genes associated with lithium treatment response as a continuous outcome. The lower panel (B) shows the candidate genes associated with lithium treatment response as a categorical binary outcome. The number on the top of the bars represents the number of SNPs located in each of the candidate genes positioned along the x-axis.

**Supplementary Table 1.** Top candidate genes from gene-based association analysis (P <0.001) of ConLi^+^Gen GWAS using MAGMA (Multi-marker Analysis of GenoMic Annotation) method [^1^](#_ENREF_1).

| Chromosome | Gene symbol | Number of SNPs | P value | Lithium treatment response outcome |
| --- | --- | --- | --- | --- |
| 1 | *S100A11* | 3 | 9.7x10^-4^ | Continuous outcome: ALDA total score |
| 2 | *TMEM131* | 185 | 1.9x10^-4^ |  |
| 4 | *RBM47* | 423 | 3.5x10^-4^ |  |
| 4 | *ELOVL6* | 306 | 7.0x10^-5^ |  |
| 4 | *ALPK1* | 281 | 3.5x10^-4^ |  |
| 7 | *TMEM196* | 84 | 2.7x10^-4^ |  |
| 7 | *ERVW-1* | 36 | 9.9x10^-4^ |  |
| 7 | *FAM133B* | 43 | 9.4x10^-4^ |  |
| 9 | *TTC39B* | 329 | 4.0x10^-4^ |  |
| 9 | *TOR1B* | 27 | 7.7x10^-4^ |  |
| 10 | *TYSND1* | 55 | 1.0x10^-4^ |  |
| 12 | *OR2AP1* | 17 | 7.8x10^-4^ |  |
| 12 | *ADGRD1* | 474 | 9.2x10^-4^ |  |
| 15 | *BMF* | 39 | 6.0x10^-5^ |  |
| 16 | *GRIN2A* | 1085 | 9.1x10^-4^ |  |
| 16 | *MYLK3* | 5 | 6.6x10^-4^ |  |
| 16 | *C16orf87* | 1 | 4.7x10^-4^ |  |
| 17 | *TMEM98* | 24 | 8.1x10^-4^ |  |
| 17 | *CDK3* | 26 | 8.8x10^-4^ |  |
| 19 | *GNG8* | 18 | 5.2x10^-4^ |  |
| 20 | *OCSTAMP* | 29 | 5.1x10^-4^ |  |
| 20 | *SLC13A3* | 175 | 7.7x10^-4^ |  |
| 1 | *CSMD2* | 1166 | 8.1x10^-4^ | Categorical outcome: ALDA≥7 |
| 1 | *SLC9C2* | 167 | 7.7x10^-4^ |  |
| 1 | *DNAH14* | 1105 | 4.4x10^-4^ |  |
| 6 | *ZBTB2* | 55 | 1.6x10^-4^ |  |
| 8 | *MTSS1* | 434 | 8.8x10^-4^ |  |
| 9 | *TOR1B* | 27 | 4.4x10^-4^ |  |
| 9 | *TOR1A* | 43 | 6.8x10^-4^ |  |
| 10 | *TYSND1* | 55 | 6.0x10^-5^ |  |
| 10 | *RNLS* | 448 | 3.0x10^-4^ |  |
| 10 | *FANK1* | 232 | 5.4x10^-4^ |  |
| 12 | *FGD4* | 735 | 1.7x10^-4^ |  |
| 13 | *RGCC* | 44 | 2.0x10^-5^ |  |
| 15 | *BMF* | 39 | 1.8x10^-4^ |  |
| 16 | *CHP2* | 14 | 5.8x10^-4^ |  |
| 17 | *TRAF4* | 12 | 8.6x10^-4^ |  |
| 17 | *TNRC6C* | 118 | 8.0x10^-5^ |  |
| 19 | *ZFP28* | 65 | 1.5x10^-4^ |  |
| 20 | *OCSTAMP* | 29 | 8.4x10^-4^ |  |
| 22 | *CRYBB3* | 49 | 3.9x10^-4^ |  |

**References**

1. de Leeuw CA, Mooij JM, Heskes T, Posthuma D. MAGMA: generalized gene-set analysis of GWAS data. *PLoS Comput Biol* 2015; **11**(4)**:** e1004219.
